# Genuine Enterprise

**Published:** 1876-01

**Authors:** 


### GENUINE ENTERPRISE.

The *Gazette*, a spicy little weekly paper in magazine form, published by Geo. P. Rowell & Co., has a recent article of a very readable character, concerning great advertisers. Among these, Dr. Dundas Dick, the manufacturer of Tasteless Medicines, comes in for a personal description, which we are inclined to copy:—

"When I reached the outside of Mr. Dundas Dick's warehouse, I found it very unlike what one would expect the exterior of a great advertiser's place of business to be. There was no ostentatious display, no large announcements of the business carried on within; simply the small, unpretentious sign on the door-post, in white letters on a blue ground, "Dundas Dick & Co." I soon made my way to the principal, and in appearance found him the *beau ideal* of a business man. His face was spare and sallow, a heavy dark moustache giving it an appearance of firmness. His manner was slightly brusque, and his readiness of apprehension testified that his quick eyes and intellectual forehead were correct indices to leading traits of his character. He was in his shirt-sleeves, and wore an apron.

"'When I started this business of capsules and tasteless medicines,' said he, 'all there was to favor the enterprise was an open field. I thought there was a prospect of doing a paying business, but the event has demonstrated that I quite underestimated the probable demand. I, of course, began in a small way, but took the precaution to invest as much as I could spare in making known through advertisements that I meant doing business. The publicity had the effect I calculated on, and in the first year my profits paid well. Every year I increased the volume of advertising, and every year the income traceable to it has increased in proportion.'"

We know Dr. Dick reasonably well, and do not wonder at his enormous success. He attends strictly to his business, and is the hardest-worked man about his establishment. He is at his factory from early morning till late at night, and there is hardly a grain of medicine leaving his establishment in its tasteless clothing, which has not more than once passed under his eye in the course of its prepation. He trusts nothing wholly to his employees; and if he had the arms of Briarius, we believe he would manage to accomplish every detail of his work unaided. He has not a helper who has not been instructed in his department by Dr. Dick in person. Like Mr. A. T. Stewart, there is no part of his business which he is not competent to do as well as any one could do it. These qualities, added to the wide publicity which he gives to his method of sealing up from injury and contamination, while depriving of taste, some potent balsamic and oleaginous medicines, have made him successful, powerful, and rich.

